# Novel Small-Molecule AMP-Activated Protein Kinase Allosteric Activator with Beneficial Effects in *db/db* Mice

**DOI:** 10.1371/journal.pone.0072092

**Published:** 2013-08-20

**Authors:** Li-Na Zhang, Lei Xu, Hua-Yong Zhou, Ling-Yan Wu, Yuan-Yuan Li, Tao Pang, Chun-Mei Xia, Bei-Ying Qiu, Min Gu, Tian-Cheng Dong, Jing-Ya Li, Jing-Kang Shen, Jia Li

**Affiliations:** National Center for Drug Screening, Shanghai Institute of Materia Medica, Chinese Academy of Sciences, Shanghai, China; Max Delbrueck Center for Molecular Medicine, Germany

## Abstract

AMP-activated protein kinase (AMPK) is an energy sensor of metabolism that is an attractive therapeutic target for type 2 diabetes mellitus and metabolic syndrome. Using a homogeneous scintillation proximity assay (SPA), we identified a new small-molecule AMPK activator, ZLN024, which allosterically stimulated active AMPK heterotrimers and the inactive α1 subunit truncations α1 (1–394) and α1 (1–335) but not α1 (1–312). AMPK activation by ZLN024 requires the pre-phosphorylation of Thr-172 by at least one upstream kinase and protects AMPK Thr-172 against dephosphorylation by PP2Cα. ZLN024 activated AMPK in L6 myotubes and stimulated glucose uptake and fatty acid oxidation without increasing the ADP/ATP ratio. ZLN024 also activated AMPK in primary hepatocytes, decreased fatty acid synthesis and glucose output. Treatment of *db/db* mice with 15 mg/kg/day ZLN024 improved glucose tolerance; liver tissue weight, triacylglycerol and the total cholesterol content were decreased. The hepatic transcriptional level of G6Pase, FAS and mtGPAT were reduced. The transcription of genes involved in fatty acid oxidation and the mitochondrial biogenesis of muscle tissue were elevated. The ACC phosphorylation was increased in muscle and liver. This study provides a novel allosteric AMPK activator for functional study *in vitro* and *in vivo* and demonstrates that AMPK allosteric activators could be a promising therapeutic approach for type 2 diabetes mellitus and metabolic syndrome.

## Introduction

Type 2 diabetes mellitus and metabolic syndrome have become an increasing health risk. Novel agents that ameliorate insulin resistance and hyperglycemia need to be explored. AMPK activators are emerging as a promising therapeutic target for type 2 diabetes mellitus and metabolic syndrome. AMPK is a highly conserved serine/threonine protein kinase that serves as an energy sensor in metabolism. It is activated under conditions that induce stress, such as exercise, ischemia, hypoxia and glucose deprivation, which are accompanied by an increasing cellular AMP/ATP or ADP/ATP ratio. AMPK is also regulated by cytokines including leptin, adiponectin, resistin, ghrelin, interleukin-6 and ciliary neurotrophic factor (CNTF). Once activated, AMPK stimulates catalytic pathways that generate ATP, such as glucose uptake and fatty acid oxidation, whereas it inhibits ATP-consuming anabolic pathways, including the synthesis of hepatic triacylglycerol, cholesterol, protein and glycogen [1], [2], [3], [4], [5].

AMPK is a heterotrimer that consists of a catalytic α subunit and two regulatory subunits, β and γ, which have multiple isoforms (α1, α2; β1, β2; γ1, γ2, γ3) and are expressed differently in various tissues and subcellular locations. The heterotrimeric complex is required for maximum enzymatic activity [6], [7]. The phosphorylation of Thr-172, which is located in the activation loop, is critical and essential for AMPK heterotrimers [8], [9]. Three kinases have been reported to act upstream of AMPK and are responsible for Thr-172 phosphorylation: Liver kinase B 1 (LKB1), which is the predominant upstream kinase in most tissues such as the liver and muscle; calcium/calmodulin-dependent protein kinase kinase β (CaMKKβ), which phosphorylates and activates AMPK *in vitro* and *in vivo* [6], [10], [11], [12], [13]; and transforming growth factor-β-activated kinase 1 (TAK1), which can phosphorylate AMPK in cell-free assays, although the activation has not been confirmed *in vivo* [14].

There are several small-molecule allosteric AMPK activators, for example, A-769662, which was identified by Abbott [15], and salicylate [16], a plant product has been in medicinal use since ancient times. A-769662 activates the AMPK heterotrimer with micromolar activity and exerts anti-diabetic effects in *ob/ob* mice. However, it only activates AMPK heterotrimers containing the β1 isoform [17] and retained its glucose-lowering effect independent of AMPK activation in hepatocytes [18], [19]. Salicylate binds at the same site as A-769662. However, besides allosteric activation, it can uncouple mitochondrial respiration to activate AMPK. And it also inhibits prostanoid biosynthesis and the I kappa B kinase beta (IKKβ) in the NF-kappa B pathway [16]. These results highlight the importance of developing novel AMPK allosteric activators for *in vivo* efficacy and functional study.

We identified ZLN024 as a novel AMPK allosteric activator that has no effect on mitochondrial function or the ADP/ATP ratio. ZLN024 provided metabolic benefits in L6 myotubes and primary hepatocytes by activating AMPK, and it reduced glucose intolerance and fatty liver characteristics in diabetic *db/db* mice. Our results suggest that this novel AMPK allosteric activator may represent a promising therapeutic approach for treating type 2 diabetes mellitus and metabolic syndrome.

## Materials and Methods

### Scintillation Proximity Assay (SPA)

Before the SPA assay, 200 nmol/l recombinant AMPK protein (α1β1γ1, α2β1γ1, α1β2γ1, α2β2γ1, α1(1–394), α1(1–335), α1(1–312)) was constructed, expressed, purified and fully phosphorylated as described previously [20]. The SPA reactions were performed in 96-well plates in a final volume of 50 µl containing 20 mmol/l Tris-HCl, pH 7.5, 5 mmol/l MgCl_2_, 1 mmol/l DTT, 2 µmol/l biotin-SAMS, 2 µmol/l ATP and 7.4×10^3^ Bq/well [γ-^33^P]ATP. The reactions were initiated by the addition of 50 nmol/l recombinant AMPK protein to the reaction solutions, followed by incubation at 30°C for 2 hr. The reactions were then terminated by the addition of 40 µl of stop solution containing 80 µg streptavidin-coated SPA beads per well, 50 mmol/l EDTA and 0.1% Triton X-100 in PBS, pH 7.5, followed by incubation for 1 hr. Finally, 160 µl of suspension solution containing 2.4 mol/l CsCl, 50 mmol/l EDTA and 0.1% Triton X-100 in PBS, pH 7.5, was added to the reaction solution to suspend the SPA beads completely. The SPA signals were measured in a Wallac Microbeta plate counter (PerkinElmer, Waltham, MA, USA) 30 min later.

### Measurement of PP2Cα Activity

Recombinant human protein phosphatase-2Cα (PP2Cα) was obtained from Abcam (Cambridge, UK). PP2Cα activity was measured using pNPP as a substrate. The activity was measured in a SpectraMAX 340 plate reader at 37°C and 410 nmol/l for 120 sec. The assay was carried out in a 96-well plate in a final volume of 100 µl containing 50 mmol/l Tris-HCl, pH 7.6, 2 mmol/l DTT, 10 mmol/l MnCl_2_ and 10 mmol/l pNPP; the PP2Cα enzyme concentration was 400nmol/l.

### Measurement of Fatty Acid Oxidation

The assay was initiated by adding [9,10-^3^H(N)]-palmitic acid (PerkinElmer, Waltham, MA, USA) to a final concentration of 250 µmol/l and 5.55×10^4^ Bq per well in DMEM. After incubation with the compound for 4 hr in differentiated L6 myotubes, sample from each well was added to charcoal slurry for centrifugation and then the radioactivity was measured.

### Measurement of Fatty Acid Synthesis

Fatty acid synthesis in rat primary hepatocytes was assayed by measuring [^14^C] acetate incorporation into lipids. Rat hepatocytes were treated for 20 hr with ZLN024 at the concentrations described in FBS-free DMEM with or without 10 nmol/l insulin, followed by 4 hr incubation in fresh medium containing 0.1 µCi/ml sodium [^14^C] acetate (3.7×103 Bq/ml). Thereafter, cells were washed three times by ice-cold PBS and analyzed as previously described [21], [22].

### Adenovirus Infection

Recombinant adenovirus expressing dominant negative forms of AMPK, AMPKα1 (D159A) and AMPKα2 (K45R) (α1/α2-DN), were constructed by using pAdEasy system (Agilent Technologies, New York, CA, USA). L6 myotubes were infected with adenovirus expressing control GFP reporter protein or α1/α2-DN, at the 4th day after differentiation before compound treatment.

### Animal Studies

C57BKS *db/db* (Jackson Laboratory, BarHarbor, ME, USA) mice were bred at the Shanghai Institute of Materia Medica (Chinese Academy of Sciences, Shanghai, People’s Republic of China).The animals were maintained under a 12 hr light–dark cycle with free access to water and food. Animal experiments were approved by the Animal Care and Use Committee, Shanghai Institute of Materia Medica. At 8 weeks of age, male *db/db* mice were randomly assigned to the various treatment groups by body weight and glucose levels (n = 6–8). The treatment groups for the 5-week chronic study were as follows: vehicle (0.5% methylcellulose), ZLN024 (15 mg/kg) and metformin (250 mg/kg). The treatments were orally administered once daily. The body weights and food intake were measured daily. After 5 weeks of treatment, the mice were killed after a final dose, and the tissues were collected for further analysis.

### RNA Isolation and Real-time PCR

This assay was described previously [23]. For details on primer sequences, see Legend for Table S1.

### Dephosphorylation of AMPK

The dephosphorylation of AMPK was performed as described previously [17], [24].

### Mitochondrial Membrane Potential, Adenine Nucleotide Measurement, Glucose Uptake And Glucose Output, Western Blot, Enzymes, Metabolite Analysis and Cell Culture

These assays were described previously [23], [25].

### Statistical Analysis

The results are presented as the mean ± SEM. The differences between the two groups were analyzed using Student’s *t*-test. The differences among multiple groups were compared by one-way ANOVA, followed by a LSD comparison. *P*<0.05 was regarded as statistically significant.

## Results

### Scintillation Proximity Assay for AMPK Activator Screening and Discovery of a Novel Allosteric Activator of AMPK Heterotrimers

Because AMPK is a promising therapeutic target, the availability of simple, sensitive, and cost-saving assays suited to high-throughput screening is crucial in discovering small-molecule AMPK activators. To meet this need, we have developed a novel assay for AMPK allosteric activator screening based on scintillation proximity assay (SPA) technology, which is currently being widely applied to the screening of various kinases [26], [27], [28]. Using a traditional filter assay, the *Km* values of the biotin-SAMS and ATP substrates toward recombinant AMPK α1β1γ1 were determined to be 48 µmol/l and 37 µmol/l, respectively (data not shown), which is in accordance with previous reports [29], [30], suggesting that biotin-SAMS and recombinant AMPK heterotrimers are practical for use in assays. After a series of experimental optimization, the final assay system was established.

**Figure 1:**
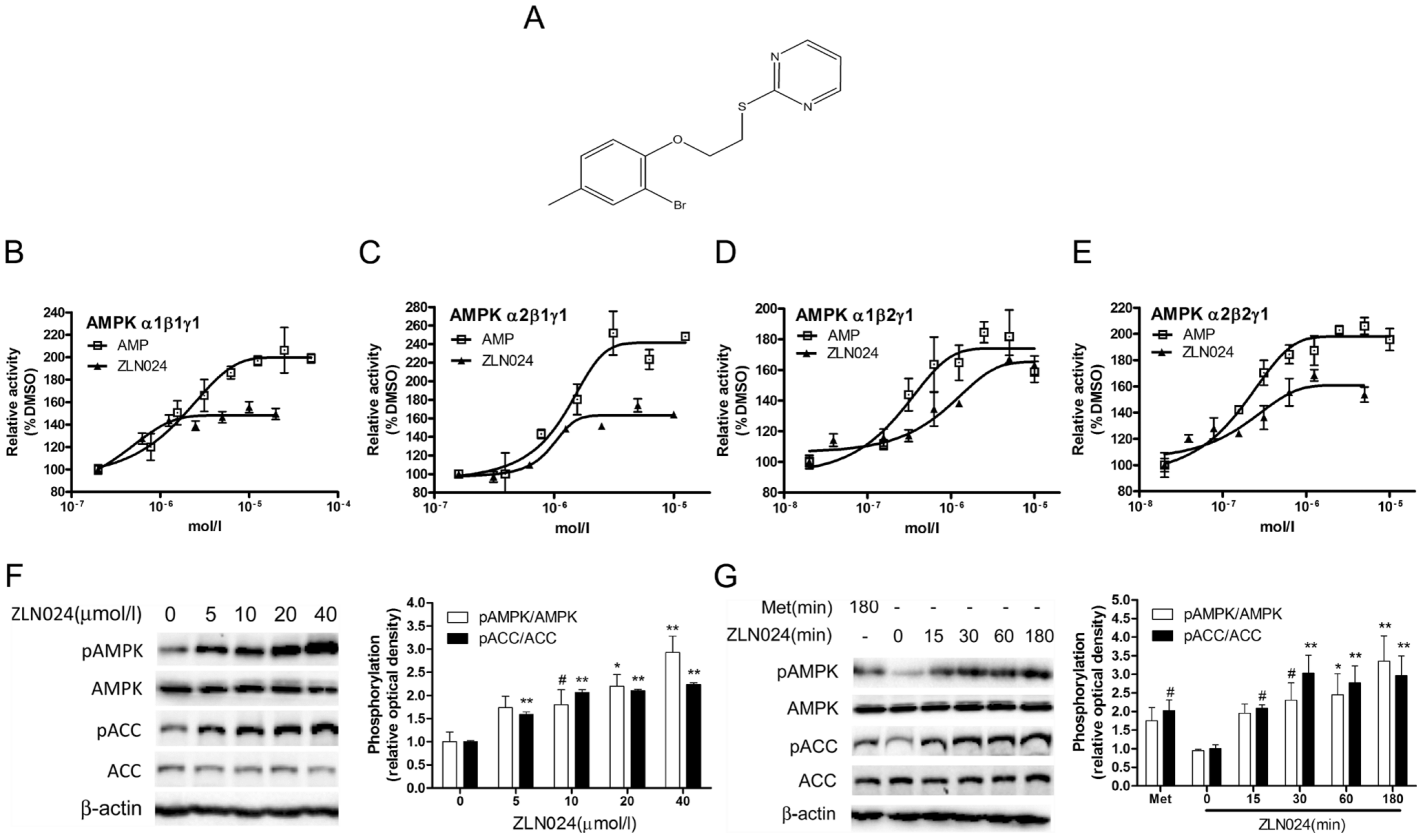
Activation of human AMPK heterotrimers by ZLN024. (A) The structure of ZLN024 (molecular weight = 325.2). (B) The activation curve for ZLN024 and recombinant human AMPK α1β1γ1 (n = 2), α2β1γ1(n = 2) (C), α1β2γ1(n = 3) (D) and α2β2γ1(n = 3) (E). (F) ZLN024 concentration-dependently stimulates AMPK and ACC phosphorylation in L6 myotubes after incubation for 3 hr. The ratio of the phosphorylation level to the protein level of AMPK and ACC was determined (n = 3). (G) The time course of the stimulation of AMPK and ACC phosphorylation by ZLN024 (20 µmol/l). Metformin (2 mmol/l) was used as a positive control. The ratio of the phosphorylation level to the protein level of AMPK and ACC was determined (n = 3). #, *P*<0.1,*, *P*<0.05, **, *P*<0.01 compared with the untreated control.

With our novel assay system, the endogenous AMPK activator, AMP, activated α1β1γ1 and α2β1γ1 by around 2–2.5 fold with an EC_50_ of about 1–2 µmol/l (Figure 1B and 1C). The activation extent of AMP on both heterotrimers was similar to published data [15], [16], [24], which might result from the different assay systems and conditions used. To date, there are two different assay methods, the traditional filter assay [31] and microarrayed compound screening (µARCS) [15], [32]. Compared with the low-throughput and laborious traditional filter assay, the µARCS assay greatly increased the throughput; however, the technique was still rather complex because of multiple procedures [15]. Our SPA assay not only is homogeneous which significantly improves the throughput of the screening, but also is suitable for quantifying the potency of the positive compound. This novel assay offers a good homogeneous assay platform for further AMPK activator discovery programs.

Using the established SPA assay, we performed random screening against the AMPK α1β1γ1 heterotrimer and found a new AMPK activator, ZLN024 (molecular weight = 325.2), with a novel structure (Fig. 1A). ZLN024 was found to directly activate recombinant AMPK α1β1γ1 and its homologue α2β1γ1 in a concentration-dependent manner. It increased the activity of α1β1γ1 by 1.5-fold and had an EC_50_ of 0.42 µmol/l, and it increased the activity of α2β1γ1 by 1.7-fold with an EC_50_ of 0.95 µmol/l (Fig. 1B–C). ZLN024 also directly activate recombinant AMPK α1β2γ1, by 1.7-fold with EC_50_ of 1.1 µmol/l; and AMPK α2β2γ1, by 1.6-fold with EC_50_ of 0.13 µmol/l (Fig. 1D–E).

After observing the allosteric activation of the AMPK heterotrimer by ZLN024, we investigated its effect in L6 myotubes. ZLN024 stimulated the phosphorylation of AMPK, and phosphorylation of the well recognized AMPK downstream Acetyl coenzyme A carboxylase (ACC) in a concentration-dependent manner (Fig. 1F). The increase of AMPK and ACC phosphorylation by ZLN024 was started within 15 min and reached maximal effect within 30 min (Fig. 1G).

### ZLN024 Allosterically Activates Human AMPK α1 Subunits and Inhibits the Dephosphorylation of AMPK Thr-172 by PP2Cα

**Figure 2:**
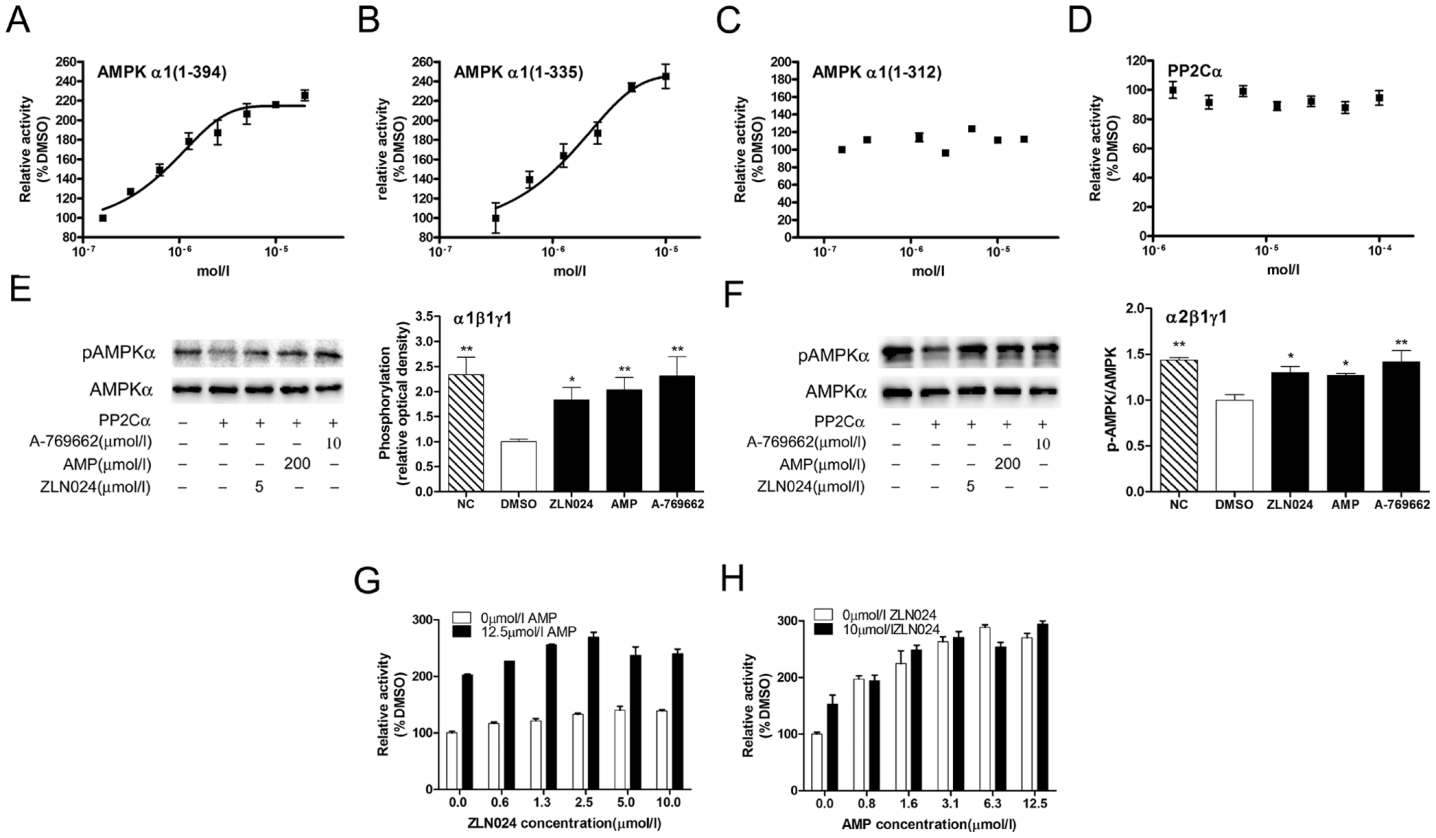
ZLN024 activates human AMPK α1 subunits and inhibits AMPK dephosphorylation by PP2Cα. (A) The activation curve for the effect of ZLN024 on the activity of the AMPKα1 truncations (1–394) (n = 2), (B) α1 (1–335) (n = 2) and (C) α1 (1–312) (n = 2). (D) The effect of ZLN024 on PP2Cα (n = 3). (E) ZLN024 inhibits AMPK α1β1γ1 (n = 5) and AMPK α2β1γ1 (n = 3) (F) dephosphorylation by PP2Cα. The ratio of the phosphorylation level to the protein level of AMPK was determined. NC = negative control, PP2Ca was not added. (G) The additive activation of AMPK α2β1γ1 by different concentrations of ZLN024 and 12.5 µmol/l AMP (n = 2). (H) The additive activation of different concentrations of AMP and 10 µmol/l ZLN024 (n = 2). #, *P*<0.1, *, *P*<0.05, **, *P*<0.01 compared with the untreated control.

We then investigated the effect of ZLN024 on the catalytic α subunit. The AMPKα1 subunit has a low basal catalytic activity when it is expressed as the full-length protein, even after pre-phosphorylation by an upstream kinase [33]. Truncation experiments revealed that the α1 subunit consists of the constitutively active catalytic domain (residues 1–312) and the autoinhibitory domain (AID) (residues 313–335) [33], [34]. To clarify the mechanism of the allosteric activation mediated by ZLN024, we examined its effect on inactive truncations of the α1 subunit containing the AID: α1(1–394) and α1(1–335), by SPA assay. ZLN024 caused a substantial activation of the two truncated proteins, with EC_50_ values of 0.92 µmol/l and 2.23 µmol/l, respectively. The maximal activation of these truncations was 2.3-fold and 2.8-fold, respectively, which was greater than the activation of the heterotrimers (Fig. 2A, B). Various concentrations of ZLN024 did not induce significant changes in the activity of the constitutively active kinase domain (KD) α1(1–312) in SPA assay (Fig. 2C).The activation of inactive α1(1–394) and α1(1–335) by ZLN024 and the inability to stimulate the activity of α1(1–312) suggest that ZLN024 may antagonize the binding of the AID (residues 313–335) to the α1 catalytic subunit and then confer a conformational change to increase the basal kinase activity of the AMPK α subunit.

The phosphorylation of Thr-172 within the catalytic α subunits is essential for the activity of AMPK [8], [9], which is phosphorylated by an upstream kinase (LKB1 or CaMKKβ) and dephosphorylated by PP2Cα [31], [35]. We investigated the ability of ZLN024 to protect against PP2Cα dephosphorylation. First, we tested the effect of varying concentrations of ZLN024 on PP2Cα activity, and no significant inhibition was observed (Fig. 2D). In contrast, the positive control (50 mmol/l NaF) inhibited the activity by 50% (data not shown). Because the AMPK conformational change elicited allosterically by ZLN024 may protect against the dephosphorylation of AMPK by PP2Cα, we further assessed the inhibitory effect of ZLN024 on the PP2Cα-catalyzed dephosphorylation of AMPK α1β1γ1 and α2β1γ1 *in vitro*. As expected, 5 µmol/l ZLN024 provided protection against dephosphorylation by PP2Cα (Fig. 2E–F); 200 mol/l AMP and 10 mol/l A-769662 inhibited dephosphorylation by PP2Cα, as previously reported [17], [24]. However, ZLN024 did not affect the dephosphorylation by PP2Cα on AMPK α1(1–394) and α1(1–335) (Fig. S1A–B).

We evaluated the additive effect of ZLN024 and AMP on AMPK α2β1γ1 stimulation. In the presence of a saturating concentration of AMP (12.5 µmol/l), increasing the concentration of ZLN024 to 10 µmol/l produced a small (1.3-fold) but significant additional activation (Fig. 2G). In the presence of a saturating concentration of ZLN024 (10 µmol/l), increasing the concentration of AMP to 12.5 µmol/l caused a significant additional activation (approximately 1.9-fold) (Fig. 2H). These results suggest that ZLN024 binds to a different site than AMP, which binds to the regulatory γ subunit. As shown in Fig. 2H, no significant additional stimulation was exhibited beyond the maximum activation of AMPK by AMP in the presence or absence of additional ZLN024, which suggests that the activation of AMPK by AMP is more potent than by ZLN024.

### ZLN024 has No Effect on the ADP/ATP Ratio in L6 Myotubes and Requires at Least One Upstream Kinase

**Figure 3:**
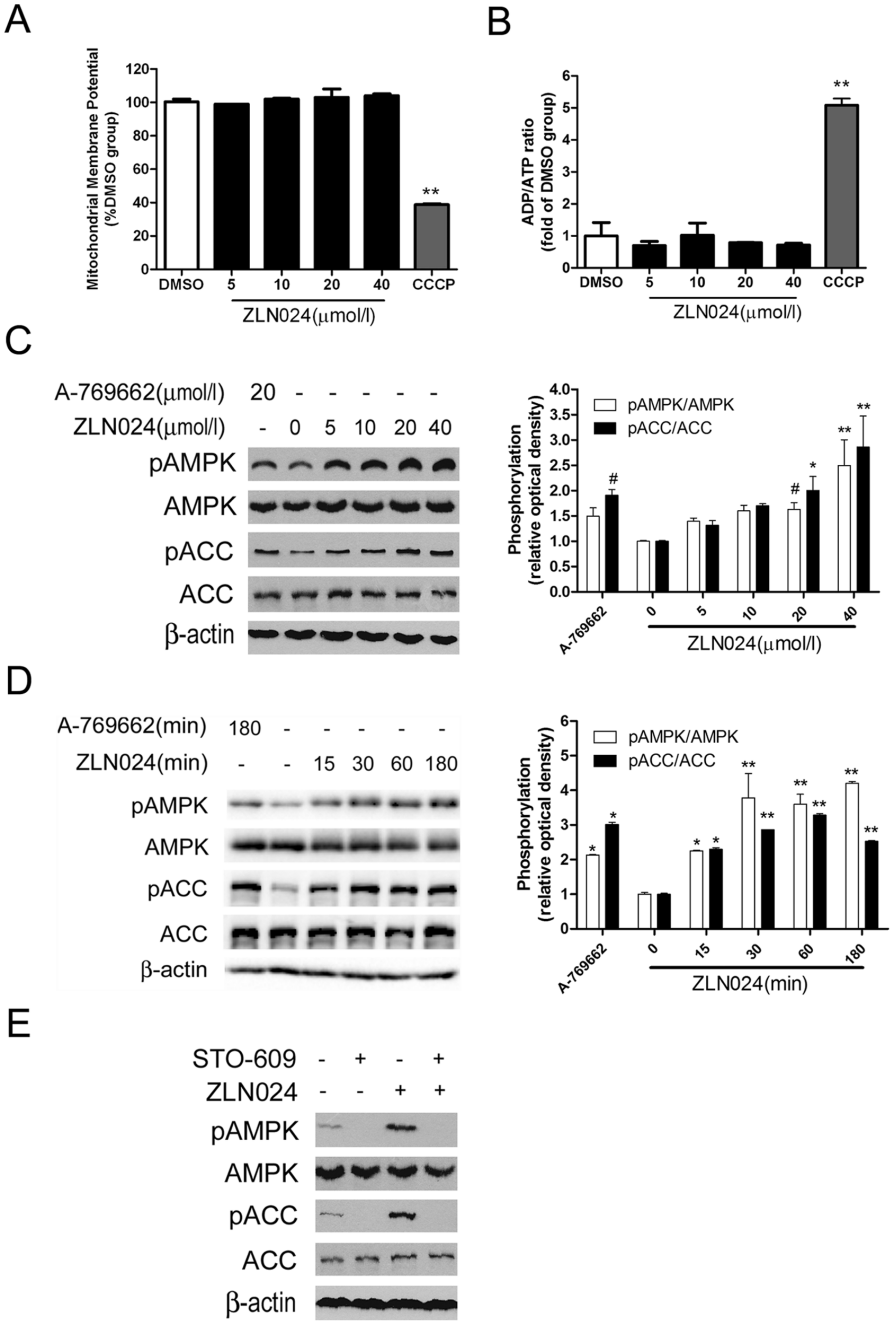
ZLN024 has no effect on the ADP/ATP ratio in L6 myotubes and requires an upstream kinase. (A) ZLN024 did not affect the mitochondrial membrane potential after incubation for 3 hr in L6 myotubes; CCCP (10 µmol/l) was used as a positive control. (B) ZLN024 did not change the ADP/ATP ratio after incubation for 3 hr in L6 myotubes, CCCP (10 µmol/l) was used as a positive control (n = 3). (C) ZLN024 stimulates AMPK and ACC phosphorylation in HeLa cells in which only CaMKKβ acts as an AMPK upstream kinase. ZLN024 was incubated for 3 hr with A-769662 (20 µmol/l) as a positive control. The ratio of the phosphorylation level to the protein level of AMPK and ACC was determined (n = 2). (D) The time course of the stimulation of AMPK and ACC phosphorylation by ZLN024 (20 µmol/l). A-769662 (20 µmol/l) was used as a positive control. The ratio of the phosphorylation level to the protein level of AMPK and ACC was determined (n = 2). (E) AMPK and ACC phosphorylation by ZLN024 is blocked by the CaMKKβ inhibitor STO609. HeLa cells were incubated with ZLN024 (20 µmol/l) for 3 hr with or without prior treatment with STO-609 (10 µg/ml) for 30 min. #, *P*<0.1, *, *P*<0.05, **, *P*<0.01 compared with the untreated control.

Changes in mitochondrial oxidative phosphorylation coupling and the cellular energy state could affect the AMP/ATP or ADP/ATP ratio, thereby leading to cellular AMPK activation [5]. The mitochondrial membrane potential (MMP) is an index of mitochondrial function, and changes in the MMP may reflect the state of energy production. To exclude an effect of ZLN024 on mitochondrial function, we investigated the effect of ZLN024 on the MMP and the AMP, ADP and ATP content in L6 myotubes. As a result, the AMP levels were too low to be reliably measured using the HPLC analysis system available to us; however, because the cellular AMP/ATP ratio varies as the square of the ADP/ATP ratio [36], we could use the ADP/ATP ratio as a surrogate measurement. As shown in Fig. 3A–B, the MMP and ADP/ATP ratio were not affected by various concentrations of ZLN024 administered for 3 hr when compared with the control, DMSO. However, carbonyl cyanide *m*-chlorophenylhydrazone (CCCP), which is a mitochondrial chemical uncoupler, decreased the MMP and increased the ADP/ATP ratio. These results suggest that ZLN024 does not interfere with mitochondrial function, and it increases the phosphorylation of AMPK by a direct allosteric interaction and not through the ADP/ATP ratio.

LKB1 and CaMKKβ have been identified as AMPK upstream kinases in cells, and the predominant upstream kinase in L6 myotubes is LKB1. To investigate the requirement for the upstream kinase in cellular AMPK stimulation by ZLN024, we utilized HeLa cells that were deficient in LKB1. ZLN024 stimulated the phosphorylation of AMPK and ACC in HeLa cells in a concentration-dependent manner after ZLN024 treatment for 3 hr (Fig. 3C); the increase of AMPK and ACC phosphorylation by ZLN024 was provoked within 15min and reached peak within 30 min (Fig. 3D). ZLN024 activated the cellular AMPK pathway independently of LKB1. To address this issue further, we performed the experiment in the presence of STO-609 in HeLa cells to block the activity of the only upstream kinase, CaMKKβ. As indicated in Fig. 3E, STO-609 completely blocked the effects of ZLN024 on the activation of AMPK. This result suggests that the allosteric activation of AMPK by ZLN024 in HeLa cells is independent of the upstream kinases LKB1 and CaMKKβ, but requires at least one upstream kinase.

### ZLN024 Stimulates Glucose Uptake and Fatty Acid Oxidation in L6 Myotubes

**Figure 4:**
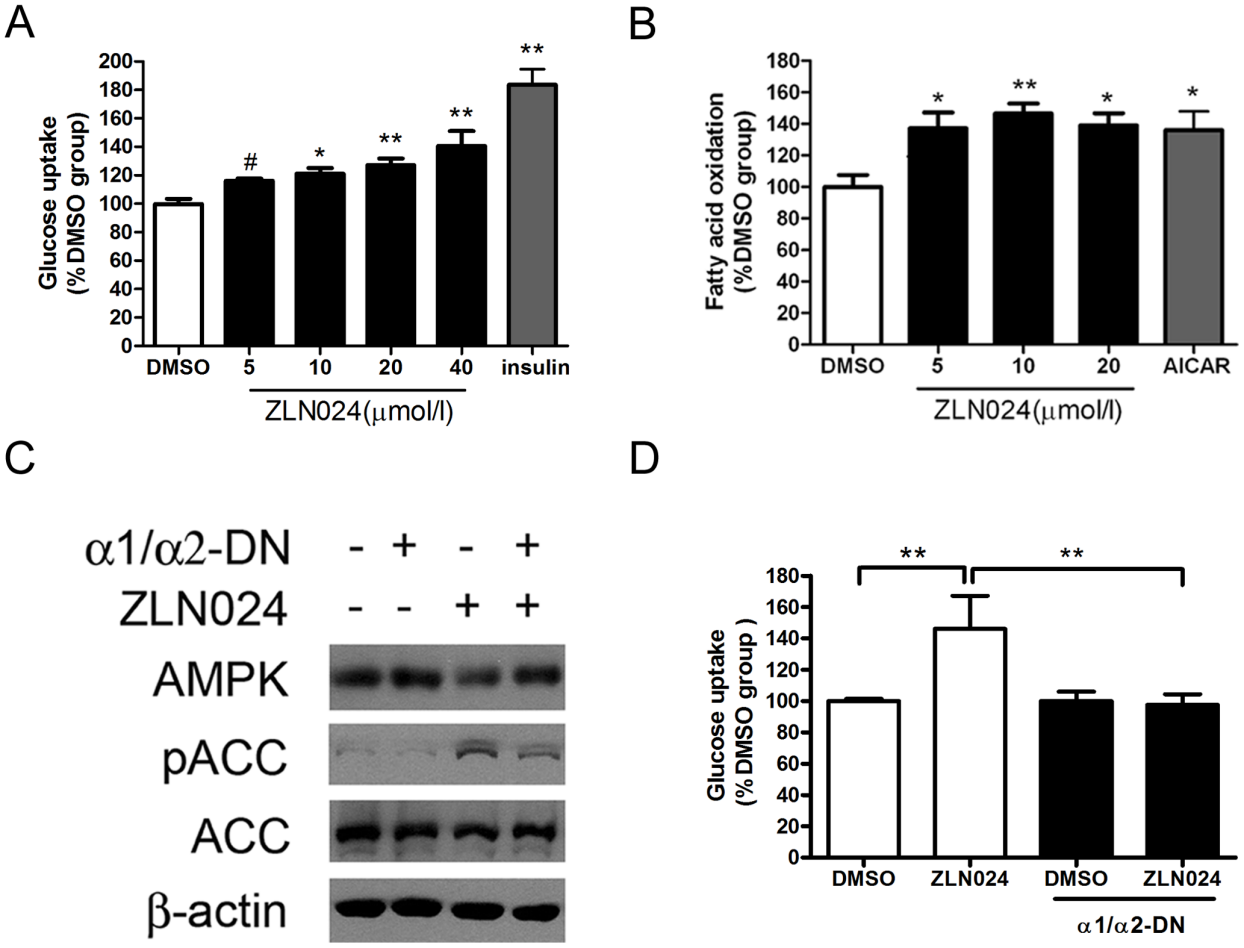
ZLN024 stimulates glucose uptake and fatty acid oxidation in L6 myotubes. (A) ZLN024 increases glucose uptake in L6 myotubes after incubation for 3 hr. Insulin (100 nmol/l) was added for the final 30 min of the experiment (n = 4). (B) ZLN024 increases fatty acid oxidation in L6 myotubes after incubation for 4 hr. AICAR (1 mmol/l) was used as a positive control (n = 3). (C) Effects of AMPK α1/α2-DN adenovirus expression on ACC phosphorylation and glucose uptake (D) caused by ZLN024 (20 µmol/l) (n = 4). #, *P*<0.1, *, *P*<0.05, **, *P*<0.01 compared with the untreated control.

Because we demonstrated that ZLN024 activates AMPK and its pathway in L6 myotubes, and the activation of AMPK in muscle has been hypothesized to stimulate glucose uptake and fatty acid oxidation [37], we further assessed these effects. As expected, the treatment of L6 myotubes with ZLN024 for 3 hr stimulated glucose uptake in a concentration-dependent manner (Fig. 4A). ZLN024 also stimulated palmitate oxidation by 40% in L6 myotubes after 4 hr treatment (Fig. 4B). We next used AMPK α1/α2-dominant negative (α1/α2-DN) adenovirus to determine the requirement for AMPK activity by ZLN024.ZLN024 activated the cellular AMPK activity was partially blocked and glucose uptake also can be blocked by infected with AMPK α1/α2-DN adenovirus (Fig. 4C, D). These findings suggest that the AMPK is involved in the stimulation of fatty acid oxidation and glucose uptake by ZLN024.

### Efficacy of ZLN024 in Primary Hepatocytes

**Figure 5:**
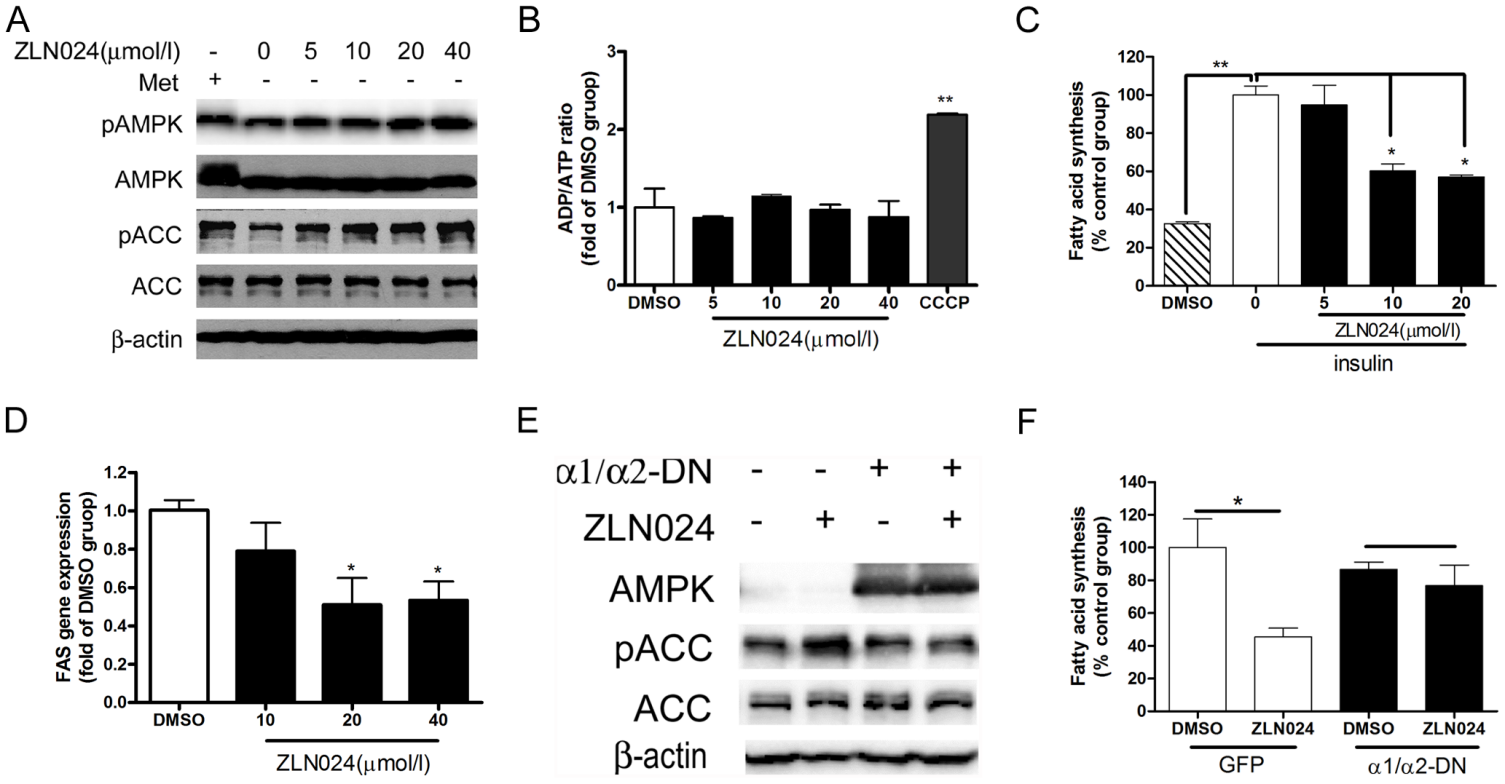
The effects of ZLN024 in rat primary hepatocytes. (A) Concentration response of the effects on AMPK and ACC phosphorylation due to ZLN024 treatment for 3 hr in rat primary hepatocytes. (B) ZLN024 did not change the ADP/ATP ratio after incubation for 3 hr in rat primary hepatocytes, CCCP (1µmol/l) was used as a positive control (n = 3). (C) Inhibition of insulin-stimulated (10 nmol/l) fatty acid synthesis by 24 hr of exposure to ZLN024. (D) The effects of treatment with ZLN024 for 21 hr on the gene expression of FAS (n = 3). (E) AMPK α1/α2-DN adenovirus expression reversed ACC phosphorylation and fatty acid synthesis (n = 2) (F) caused by ZLN024 (20 µmol/l) effectively for 24 hr treatment (n = 2). *, *P*<0.05, **, *P*<0.01 compared with the untreated control.

Since activation of AMPK is known to regulate lipid and glucose metabolism in liver, the effects of ZLN024 on fatty acid synthesis and gluconeogenesis were evaluated in primary rat hepatocytes [1], [38], [39]. First, ZLN024 slightly activated the phosphorylation of AMPK in hepatocytes and the phosphorylation of ACC was obviously increased (Fig. 5A). As shown in Fig. 5B, the ADP/ATP ratio in hepatocytes was not affected by ZLN024 as in L6 myotubes. As shown in Fig. 5C–D, ZLN024 significantly decreased the insulin stimulated synthesis of fatty acid and gene expression of fatty acid synthase (FAS). Moreover, the promotion ACC phosphorylation and the suppression of fatty acid synthesis by ZLN024 were blocked by AMPK α1/α2-DN adenovirus pretreatment (Fig. 5E–F), suggesting that ZLN024 inhibited lipid synthesis by activating AMPK.

We next evaluated the glucose production and expression of gluconeogenic genes. As expected, glucose production was obviously repressed by ZLN024 in a concentration-dependent manner (Fig. S2A). After a 21 hr incubation of primary hepatocytes with ZLN024, the mRNA levels of phosphoenolpyruvate carboxykinase (PEPCK) and Glucose 6-phosphatase (G6Pase) decreased significantly (Fig. S2B).

### Chronic Effects of ZLN024 in db/db Mice

**Figure 6:**
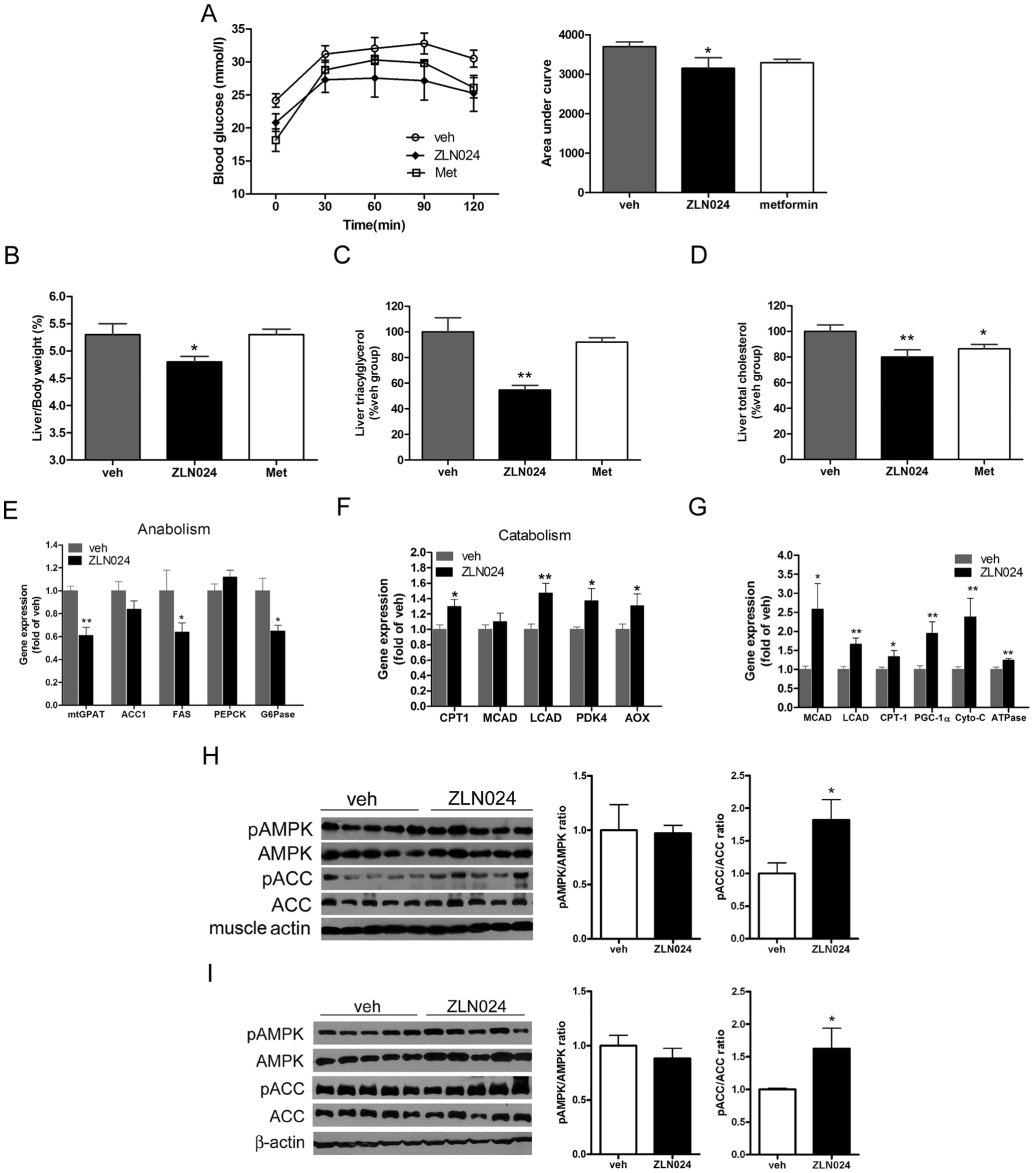
Chronic effects of ZLN024 in *db/db* mice. Eight-week-old male *db/db* mice were gavaged with vehicle (0.5% methylcellulose), ZLN024 (15 mg/kg/day) or metformin (250 mg/kg^−/^day) (*n* = 6–8). (A) Blood glucose levels after an intra-peritoneal glucose load (1.5 g/kg) performed after 4 weeks of treatment. The AUC were used as an indicator of glucose clearance. White circles, vehicle; black circles, ZLN024; white squares, metformin. (B) Amount of liver tissue in proportion to body weight (wt/wt) after 5 weeks of treatment. (C) Liver triacylglycerol level after 5 weeks of treatment. (D) Liver total cholesterol level after 5 weeks of treatment. (E) Relative anabolic gene expression levels in the liver. (F) Relative catabolic gene expression levels in the liver. (G) Relative gene expression levels in abdominal muscle. (H) AMPK and ACC phosphorylation levels in the abdominal muscle. (I) AMPK and ACC phosphorylation levels in the liver. *, *P*<0.05, **, *P*<0.01 compared with vehicle.

The beneficial effects observed at the cellular level after ZLN024 treatment prompted us to further evaluate its efficacy *in vivo*. C57BKS *db/db* mice were administered a 15 mg/kg/day dose of ZLN024 by daily gavage for 5 weeks; 250mg/kg/day metformin (Met) was used as a positive control. During the treatment period, there was no significant alteration in food intake and body weight compared with the vehicle group (Fig. S3A, B). After 4 weeks of treatment, ZLN024 improved glucose tolerance, as evidenced by the reductions of approximately 15% in the AUC (Fig. 6A). ZLN024 reduced the fasting blood glucose by 15% but not significantly with ANOVA statistic (Fig. S3C), meanwhile, metformin reduced it significantly by 24%.

Fatty liver always accompanies obesity and type 2 diabetes; excessive triacylglycerol accumulation in the liver tissue increases the liver weight and deteriorates hepatic insulin resistance. Treatment with ZLN024 markedly decreased the liver weight, as indicated by a liver/body weight reduction of 9% (wt/wt) (ZLN024 vs. vehicle, 4.8% vs. 5.3%), whereas the metformin group exhibited no decrease (Fig. 6B). Because the liver weight decreased upon ZLN024 treatment, we next investigated the hepatic triacylglycerol and total cholesterol content. As shown in Fig. 6C, ZLN024 reduced the liver triacylglycerol levels by approximately 45%, whereas an effect of metformin was not obvious. Total cholesterol levels decreased by 20% and 14% in the ZLN024 and metformin groups, respectively (Fig. 6D).

AMPK has been speculated to activate catabolic pathways that generate ATP while deactivating anabolic pathways that consume ATP [40]. To investigate the observed improvement in glucose and lipid metabolism, we analyzed the expression of genes involved in the catabolic and anabolic pathways in the liver. ZLN024 treatment significantly reduced the expression of anabolic genes, such as FAS, mitochondrial glycerol-3-phosphate acyltransferase (mtGPAT) and G6Pase; interestingly, the expression of PEPCK remained unchanged (Fig. 6E). The expression of catabolic genes, such as carnitine palmitoyl transferase I (CPT1), long chain acyl CoA dehydrogenase (LCAD), pyruvate dehydrogenase 4 (PDK4) and alternative oxidase (AOX), were mildly increased (Fig. 6F).

We next analyzed the expression of genes related to fatty acid oxidation and mitochondrial biogenesis in the abdominal muscle. ZLN024 treatment significantly upregulated genes related to fatty acid oxidation, such as CPT-1, medium chain acyl CoA dehydrogenase (MCAD) and LCAD. Genes involved in mitochondrial biogenesis, such as peroxisome proliferators activated receptor γ coactivator α (PGC-1α), cytochrome c (cyto-c) and ATPase (Fig. 6G), were also upregulated after ZLN024 treatment. These results demonstrate that ZLN024 likely ameliorates excessive lipid accumulation by increasing fatty acid oxidation and decreasing lipid synthesis.

We further measured AMPK and ACC phosphorylation in abdominal muscle and liver tissue of *db/db* mice. AMPK phosphorylation was not affected; however, ACC phosphorylation was increased in both tissues (Fig. 6H, I). These results suggested that ZLN005 activated AMPK pathway in *db/db* mice.

## Discussion

Attention has been focused on AMPK because of its important roles in the regulation of metabolism. Metformin and TZDs are drugs that are widely prescribed for the treatment of type 2 diabetes mellitus and exert their effects partly through the activation of AMPK [41], [42], [43], [44]. Natural products such as berberine, resveratrol, epigallocatechin and capsaicin also exert glucose-lowering effects, partly by activating AMPK through the regulation of mitochondrial function [3], [44]. These compounds mainly activate AMPK indirectly by increasing the AMP/ATP ratio. Because changes in the mitochondrial and cellular energy state could lead to a broad spectrum of effects and may cause side effects, direct allosteric activation of AMPK without increasing the cellular AMP/ATP ratios represents a novel therapeutic approach for treating metabolic syndrome.

AMPK activators with different mechanisms have been reported. 5-Amino-imidazole carboxamide ribonucleotide (AICAR) [45], [46], [47], [48] activates AMPK upon its conversion to ZMP, which mimics the effects of AMP on AMPK that involve the allosteric modulation and inhibition of dephosphorylation by PP2Cα [49]. A-769662 and salicylate activates AMPK through an allosteric activation that is dependent on the glycogen-binding domain (GBD) within the β1 subunit and the inhibition of dephosphorylation by PP2Cα [17], [24]. ZLN024 directly activates the AMPK heterotrimers α1β1γ1, α2β1γ1, α1β2γ1 and α2β2γ1; it can also antagonize the autoinhibition of the α1 subunit, as evidenced by the activation of the inactive truncations α1(1–394) and α1(1–335), as well as the inability to activate the active truncation α1(1–312). These results suggest that the binding site of ZLN024 is located in the α1 subunit and that the subsequent conformational change may relieve the autoinhibition, thereby activating AMPK. The conformational change conferred by ZLN024-mediated activation was further confirmed by the ZLN024-associated protection against Thr-172 dephosphorylation by PP2Cα. ZLN024 had no effect on the enzymatic activity of PP2Cα. Similarly to A-769662, salicylate and AMP, ZLN024 activates AMPK by allosteric activation and the inhibition of dephosphorylation. Since the mild activation of ZLN024 on AMPK heterotrimers (about 1.5-fold), we supposed the protection of dephosphorylation by PP2Cα might play important role in the biological effects of ZLN024 in cellular level.

To further investigate the potential mechanism of action of ZLN024, we ruled out the potential influence of ZLN024 on the cellular ADP/ATP ratio. Many AMPK activators work by regulating mitochondrial function and reducing ATP generation, which is related to the MMP. ZLN024 had no effect on the MMP or the ADP/ATP ratio, which suggests that cellular AMPK activation occurs primarily through allosteric activation and protection of dephosphorylation. We then observed a robust stimulation of the AMPK pathway in L6 myotubes and rat primary hepatocytes by ZLN024. The stimulation mostly abrogated by treatment with the adenovirus infection of AMPK α1/α2-DN, which confirmed that the activity of ZLN024 requires a functional AMPK pathway.

Based on our *in vitro* findings, we sought to test the effects of ZLN024 *in vivo*. We assessed the chronic effects of ZLN024 in *db/db* mice. ZLN024 improved glucose homeostasis, as evidenced by an improvement in glucose tolerance. The prolonged activation of AMPK leads to increased insulin sensitivity [46], [50], primarily via effects on lipid metabolism. ZLN024 may alleviate glucose intolerance primarily through dyslipidemic control, which can be inferred from the decrease in liver weight and liver triacylglycerol content. The decrease in liver triacylglycerol may result from the increased fatty acid oxidation and decreased fatty acid synthesis mediated by AMPK, as evidenced by the mild upregulation of genes involved in fatty acid oxidation in liver and muscle, and downregulation of fatty acid synthesis in liver. Consistent with the results *in vivo*, fatty acid synthesis was reduced by ZLN024 treatment in hepatocytes.

In summary, we have identified a novel allosteric AMPK activator ZLN024, which exerts beneficial metabolic effects *in vitro* and *in vivo*. Our findings provide another tool for understanding the allosteric activation of AMPK and suggest that the direct activation of AMPK is a promising approach for discovering novel therapies for the treatment of type 2 diabetes mellitus and metabolic diseases.

## Supporting Information

Figure S1 **ZLN024 does not affect AMPK α1 truncations dephosphorylation by PP2Cα.** Effect of AMP, A-769662, ZLN024 on AMPK α1(1–394) (n = 2) (A) and AMPK α1(1–335) (n = 2) (B) dephosphorylation by PP2Cα. The ratio of the phosphorylation level to the protein level of AMPK was determined. NC = negative control, PP2Ca was not added. *, *P*<0.05, **, *P*<0.01 compared with the DMSO group.
(TIF)

Figure S2 **ZLN024 decreases gluconeogenesis in rat primary hepatocytes.** (A) Treatment with ZLN024 for 5 hr decreases glucose production in hepatocytes; metformin (1 mmol/l) was used as a positive control (n = 3). (B) The effects of treatment with ZLN024 for 21 hr on the gene expression of PEPCK and G6Pase (n = 3). #, *P*<0.1, *, *P*<0.05, **, *P*<0.01 compared with the untreated.
(TIF)

Figure S3 **Chronic effects of ZLN024 on diet, weight and fasting blood glucose in** ***db/db*** **mice.** Eight-week-old *db/db* mice were gavaged with vehicle (0.5% methylcellulose), ZLN024 (15 mg/kg/day) or metformin (250 mg/kg/day) (*n* = 6–8) for 5 weeks. (A) Body weight. (B) Food intake. (C) Fasting blood glucose levels after 4 weeks of treatment; the mice were fasted for 6 hr. #, *P*<0.1, *, *P*<0.05, **, *P*<0.01 compared with the vehicle group.
(TIF)

Table S1 **The sequences of oligonucleotide primers.** The detail primer sequences for real-time PCR, all samples were run in duplex and normalized to actin expression.
(DOCX)
