# Supplementary figures and images for: Novel Small-Molecule AMP-Activated Protein Kinase Allosteric Activator with Beneficial Effects in db/db Mice
Source: PLoS One. 2013 Aug 20;8(8):e72092. doi: 10.1371/journal.pone.0072092 (PMC3748009; doi:10.1371/journal.pone.0072092)

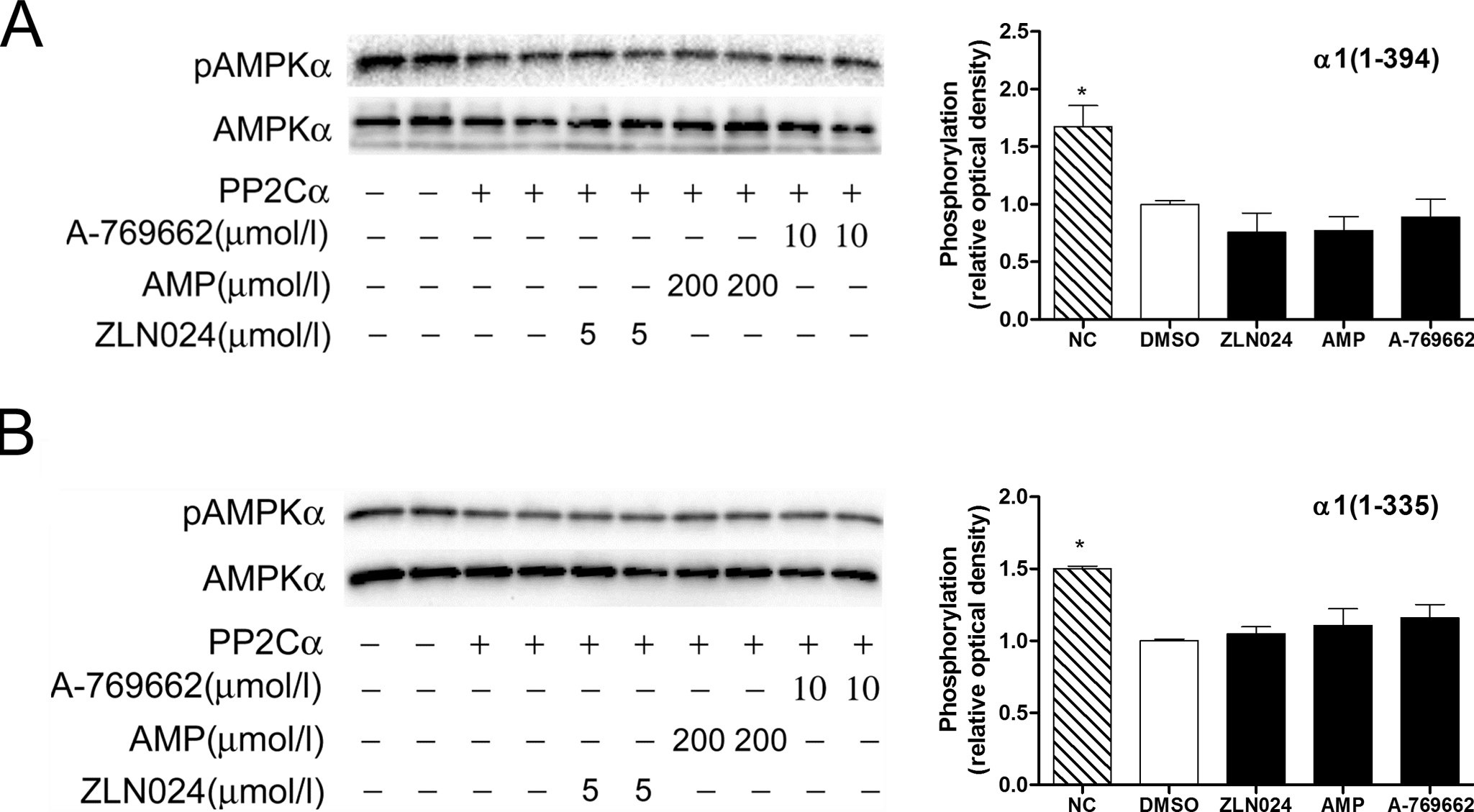

Supplement: Figure S1 — ZLN024 does not affect AMPK α1 truncations dephosphorylation by PP2Cα. Effect of AMP, A-769662, ZLN024 on AMPK α1(1–394) (n = 2) (A) and AMPK α1(1–335) (n = 2) (B) dephosphorylation by PP2Cα. The ratio of the phosphorylation level to the protein level of AMPK was determined. NC = negative control, PP2Ca was not added. *, P<0.05, **, P<0.01 compared with the DMSO group. (TIF) [file pone.0072092.s001.tif]

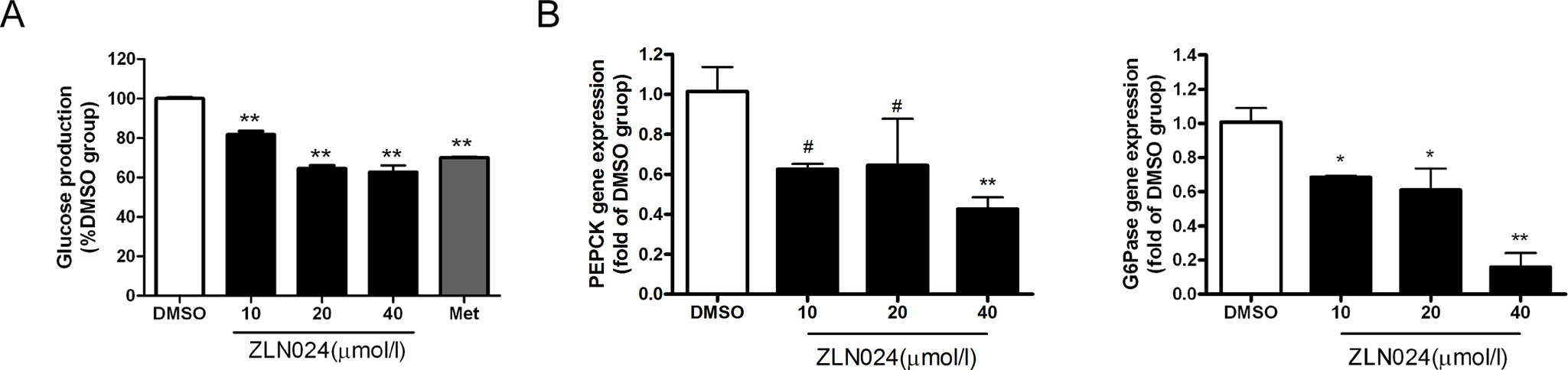

Supplement: Figure S2 — ZLN024 decreases gluconeogenesis in rat primary hepatocytes. (A) Treatment with ZLN024 for 5 hr decreases glucose production in hepatocytes; metformin (1 mmol/l) was used as a positive control (n = 3). (B) The effects of treatment with ZLN024 for 21 hr on the gene expression of PEPCK and G6Pase (n = 3). #, P<0.1, *, P<0.05, **, P<0.01 compared with the untreated. (TIF) [file pone.0072092.s002.tif]

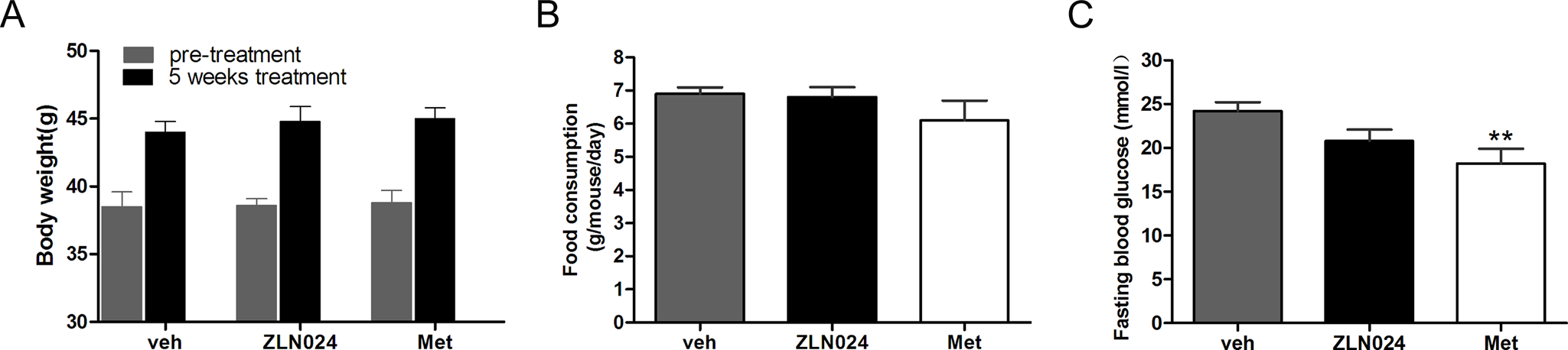

Supplement: Figure S3 — Chronic effects of ZLN024 on diet, weight and fasting blood glucose in db/db mice. Eight-week-old db/db mice were gavaged with vehicle (0.5% methylcellulose), ZLN024 (15 mg/kg/day) or metformin (250 mg/kg/day) (n = 6–8) for 5 weeks. (A) Body weight. (B) Food intake. (C) Fasting blood glucose levels after 4 weeks of treatment; the mice were fasted for 6 hr. #, P<0.1, *, P<0.05, **, P<0.01 compared with the vehicle group. (TIF) [file pone.0072092.s003.tif]
