# Supplementary material for: Novel Small-Molecule AMP-Activated Protein Kinase Allosteric Activator with Beneficial Effects in db/db Mice
Source: PLoS One. 2013 Aug 20;8(8):e72092. doi: 10.1371/journal.pone.0072092 (PMC3748009; doi:10.1371/journal.pone.0072092)
Supplement: Table S1 — The sequences of oligonucleotide primers. The detail primer sequences for real-time PCR, all samples were run in duplex and normalized to actin expression. (DOCX) [file pone.0072092.s004.docx]

| Genes | Sequence |
| --- | --- |
| actin (rat, mouse) forward | CGAGTACAACCTTCTTGCAG |
| actin (rat, mouse) reverse | GAGTCCTTCTGACCCATACC |
| PGC-1α (rat, mouse) forward | ACTGAGCTACCCTTGGGATG |
| PGC-1α (rat, mouse) reverse | TAAGAATTTCGGTGGTGACA |
| Cytochrome-C (rat, mouse) forward | GGAGGCAAGCATAAGACTGG |
| Cytochrome-C (rat, mouse) reverse | TCCATCAGGGTATCCTCTCC |
| G6Pase (rat, mouse) forward | ACACCGACTACTACAGCAACAG |
| G6Pase (rat, mouse) reverse | CCTCGAAAGATAGCAAGAGTAG |
| PEPCK (rat, mouse) forward | CATATGCTGATCCTGGGCATAAC |
| PEPCK (rat, mouse) reverse | CAAACTTCATCCAGGCAATGTC |
| FAS (rat, mouse) forward | GCTGCGGAAACTTCAGGAAAT |
| FAS (rat, mouse) reverse | AGAGACGTGTCACTCCTGGACTT |
| mouse ACC1 forward | CTTGGAGCAGAGAACCTTCG |
| mouse ACC1 reverse | ACTTCCCGACCAAGGACTTT |
| mouse AOX forward | GCTCAGCAGGAGAAATGGATGC |
| mouse AOX reverse | AATGAACTCTTGGGTCTTGGGG |
| mouse ATPase-F1α forward | TCTCCATGCCTCTAACACTCG |
| mouse ATPase-F1α reverse | CCAGGTCAACAGACGTGTCAG |
| mouse CPT-1 forward | ATGACGGCTATGGTGTTTCC |
| mouse CPT-1 reverse | TGTCCATCATGGCTTGTCTC |
| mouse MCAD forward | AGCTGCTAGTGGAGCACCAAG |
| mouse MCAD reverse | TCGCCATTTCTGCGAGC |
| mouse LCAD forward | TCACCAACCGTGAAGCTCGA |
| mouse LCAD reverse | CCAAAAAGAGGCTAATGCCATG |
| mouse PDK4 forward | ATCTAACATCGCCAGAATTAAACC |
| mouse PDK4 reverse | GGAACGTACACAATGTGGATTG |
| mouse mtGAPT forward | CAACACCATCCCCGACATC |
| mouse mtGAPT reverse | GTGACCTTCGATTATGCGATCA |
